# Is There a Relationship between Surface Wettability of Structured Surfaces and Lyophobicity toward Liquid Metals?

**DOI:** 10.3390/ma13102283

**Published:** 2020-05-15

**Authors:** Stephan Handschuh-Wang, Lifei Zhu, Tao Wang

**Affiliations:** 1 College of Chemistry and Environmental Engineering, Shenzhen University, Shenzhen 518055, China; 1800221021@email.szu.edu.cn; 2 Functional Thin Films Research Center, Shenzhen Institutes of Advanced Technology, Chinese Academy of Sciences, Shenzhen 518055, China; tao.wang1@siat.ac.cn

**Keywords:** structured diamond coating, liquid metal, diffusion barrier, surface wettability, surface roughness, adhesion

## Abstract

The liquid metal lyophobicity of a rough substrate was, in previous articles, found to be rather independent on the surface wettability. In this article, we scrutinize the impact of surface wettability of a structured (rough) surface on the liquid metal wettability and adhesion. As a model system, a structured diamond coating was synthesized and modified by air plasma. We show that surface wettability (surface free energy) does not play a prominent role for static contact angle measurements and for the liquid metal repelling properties of the diamond coating in droplet impact experiments. In contrast, roll off angles and repeated deposition experiments illustrate that the increased hydrophilicity impacts the long-term liquid metal repellency of our coating. Liquid metal adhered after around 50 deposition/removal cycles on the hydrophilic diamond coating, while no liquid metal adhesion was visible after 100 cycles on the hydrophobic diamond coating, illustrating the fundamental role for the adhesion of liquid metal. The effect of repeated deposition in conjunction with gentle applied force was employed for coating the liquid metal lyophobic (hydrophilic) diamond coating with a thin liquid metal layer. The observed effect may find application in flexible electronics and thermal management systems as a means to improve interfacing of the liquid metal with conductive non-metal coatings.

## 1. Introduction

Room temperature liquid metals, metals, and alloys liquid at or near room temperature have shown great promise as emerging materials and building blocks for a wide variety of applications, ranging from soft electronics to thermal interface materials [1,2,3,4]. The great impact of these room temperature liquid metals (for simplicity we call them in this article “liquid metals”) for soft electronics and thermal management systems is related to the atypical liquid state of the metals and alloys while possessing good electric and thermal conductivity [5]. Therefore, liquid metals are potent candidates for shaping reconfigurable and stretchable electronics, as well as adaptable electromagnetic components, such as polarizers [6] and antennas [7].

In the flexible electronics setting, liquid metals are encumbered with at least two issues, liquid metal corrosion, and poor adhesion/interfacing. First, to conduct electricity, the liquid metal needs to interface with the solid metal. However, liquid metals typically corrode solid metals, often via an alloying mechanism. Though this alloying (reactive wetting) can be employed in selective wetting/patterning methods [1,8,9,10,11,12], this alloying may lead to degradation of device performance or even to device failure [13]. To mitigate this liquid metal corrosion (liquid metal embrittlement), several approaches have been suggested. For example, coating of the liquid metal with conductive micro/nano particles was advocated as a means to avoid direct contact between liquid metal and solid metal, thus preventing liquid metal-induced corrosion of the solid metal [14]. On the other hand, conductive diffusion barriers were proposed to mitigate the corrosivity of the liquid metal toward the solid metal [15,16,17,18,19,20]. Second, a drawback of these approaches is that the liquid metal often shows poor wetting and adhesion towards the coating of the substrates. While this poor adhesion may be useful for reconfigurable electronics [21], such as antennas, it is detrimental to the performance of a device due to low contact area between the liquid metal and the coated substrate. In some recent publications, it was suggested that rendering a smooth surface hydrophilic increases the adhesion of the liquid metal (conductor), which improved the performance of liquid metal-based flexible electronic devices upon bending and stretching [22,23,24]. This effect was ascribed to enhanced adhesion due to lower surface roughness and hydrogen–bond interaction between the hydrophilic liquid metal oxide [25] and the (coated) substrate surface. However, transfer of this technique to rough surfaces, is not trivial, as the liquid metal features an oxide skin with high yield stress [26], which results in peculiar wetting and adhesion properties of the liquid metal. For example, liquid metal does not wet most surfaces and exhibits a high “non-equilibrium” (static) contact angle on these substrates. Notably, the wetting and adhesion of liquid metal is strongly dependent on the surface roughness of the substrate surface [21,27], as well as the liquid metal oxide [28]. Rough (superhydrophobic) surfaces are often found to exhibit strong liquid metal lyophobicity, enabling high mobility of the liquid metal on these surfaces [29]. Recently, a conductive composite polymer made of poly(3,4-ethylenedioxythiophene) polystyrene sulfonate (PEDOT:PSS) and graphene oxide was proposed as a diffusion barrier [15], necessitating carbon nanofiber coating of the liquid metal to enable movement of the liquid metal on the conductive composite material, as this coating is rather smooth. However, polymer coatings suffer from poor mechanical (abrasion) stability, chemical stability, and low adhesion toward the solid support (solid metal). Other properties to change the adhesion of the liquid metal are application of force, typically in so-called forced wetting approaches for liquid metal printing [30,31,32,33,34,35], loading of liquid metal with particles, so-called greases [36,37,38], and so on, while for rough surfaces, the surface free energy was found to be of subordinate importance for wetting and adhesion of a substrate with liquid metal [29].

Herein, we investigate the effect of surface chemistry (surface free energy) of a structured liquid metal repellent surface on the wetting and adhesion properties of liquid metal (oxide) combined with the aid of slight force (forced wetting). To facilitate this, we employed a structured diamond coating, which shows good liquid metal lyophobicity in the as-prepared (hydrophobic) state, as well as excellent chemical stability towards the employed liquid metal (Galinstan) [39]. The diamond coating was also chosen due to the high chemical and mechanical resistance, as well as high tuneability in terms of electrical and thermal properties [40,41], which might be investigated in future. By oxidation with air plasma, the diamond coating becomes hydrophilic while maintaining morphology and topography. The wettability and adhesion of liquid metal toward the two diamond coatings with different surface free energy (water wettability) were scrutinized by contact angle measurements, both water contact angle and liquid metal contact angle, by roll off angle measurements of liquid metal droplets, droplet impact experiments, and repeated deposition and removal experiments. Upon gentle force and repeated deposition on the oxidized diamond coating, the liquid metal was found to adhere to the diamond coating, which was employed to achieve a high contact area between the liquid metal (oxide) and the diamond coating, while this is difficult to achieve for the hydrophobic (as-prepared) diamond coating.

## 2. Materials and Methods

The titanium alloy (Ti6Al4V) substrates were cut into pieces of dimensions of 1 cm × 1 cm and underwent pretreatment, as follows. The substrates were grinded with SiC (silicon carbide) sandpaper (800, 1500, and 2000 ISO/FEPA Grit designation). Afterwards, they were cleaned ultrasonically in water and ethanol for 10 min, successively. After pre-treatment, the substrates were immersed in a diamond seeding solution. The seeding solutions comprise commercially available detonation nanodiamond (DND) particles (PL-Nanopure, grade G01, Plasmachem GmbH, Berlin Germany), aqueous solution, and oxalic acid at a final concentration of 7 × 10^−5^ mol/L. The DND particle concentration was 0.005 wt.%, while the pH was adjusted to pH 5. Subsequently, diamond was grown by hot filament chemical vapor deposition (HFCVD). The flow rates of the reaction gases CH_4_ and H_2_ were maintained at 32 sccm and 800 sccm, respectively, the temperature of the hot filaments was maintained at 2500 °C, and the gas pressure was kept at 1.5 kPa. Deposition for 1 h yielded well-distributed diamond hemispheres on the substrate. Subsequently, the substrates were oxidized and seeded again ultrasonically (15 min) with a solution comprising commercially available 0.005 wt.% DND (Chengdu Dreiway Technology, Chengdu, China) aqueous solution and 5 × 10^−6^ mol/L [2-(Methacryloyloxy)ethyl]trimethylammonium chloride (TMAEMC, 75 wt.% in H_2_O), while the pH was adjusted to 3. After a second growth step via HFCVD (same parameters) for 20 min, the diamond coatings were obtained.

Liquid metal (LM, Galinstan) was deposited on the oxidized and as-prepared diamond coating and massaged gently with the help of a soft plastic syringe. After coating of the substrate, the bulk LM was removed with another syringe. The LM was left for 4 days on top of the diamond coating. Then, the LM was removed by immersing the coated Ti alloy in 1 mol/L hydrochloric acid for 10 min and subsequently rinsed with deionized water (DI) water. The cleaned samples were subjected to microscopy, as well as SEM and EDS analysis.

Analysis: Contact angles were determined with a contact angle microscope (SDC-200, Sindin, Dongwan, China). The roll-off angle was measured employing a stage with an adjustable tilt angle (Optical Contact Angle Measuring Instrument, Sindin, China). The critical tilt angle (*θ*_c_) was determined by gradually increasing the tilt angle until the LM droplet started to move. The experiment was repeated at least five times and an average was calculated. The presence of macroscopic features of liquid metal on the diamond surface was detected by optical microscopy (SMZ18, Nikon Instruments, Shanghai, China), equipped with a complementary metal-oxide semiconductor (CMOS) camera and a digital camera (D7100, Nikon Instruments, Shanghai, China). For repeatable droplet impact experiments on the diamond coated samples, Galinstan droplets (10 µL) were generated with the help of a syringe pump outfitted with a fluorocarbon nozzle (inner diameter 1.35 mm, outer diameter 1.95 mm). The syringe was equipped to a contact angle microscope (SDC-200, Sindin, Dongwan, China) and the dispensing unit was employed to generate droplets with the same volume impacting at the same location of the sample. Impact heights of 1–24 cm were realized by changing the height of the syringe and sample. Afterwards, microscopy images (or SEM micrographs, FEI APREO S, FEI China, Shanghai, China) of the samples were taken. Scanning electron microscopy (SEM, FEI APREO S, FEI China, Shanghai, China) and energy-dispersive X-ray spectroscopy (EDS, FEI APREO S, FEI China, Shanghai, China) were measured with a FEI APREO S. Micro-Raman and scattering was measured with a Raman spectrometer (HORIBA LabRAM HR800 Evolution, HORIBA Instruments, Shanghai, China) at an excitation wavelength of 532 nm.

## 3. Results and Discussion

### 3.1. Synthesis of Hierarchical Rough Diamond Coating on Ti Alloy

**Figure 1:**
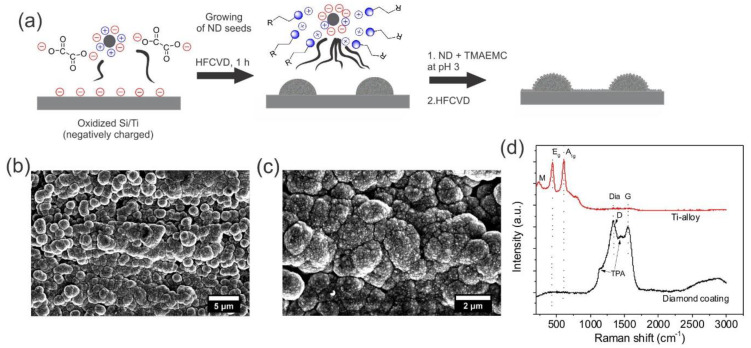
(**a**) Synthesis strategy of the as rough diamond surface via two successive seeding and growth cycles. SEM micrographs of the as-made rough diamond coating on Ti alloy (**b**) and its magnification (**c**). (**d**) Raman spectrum of the diamond coating from (**b**) and (**c**). Here, E_g_ and A_1g_ denote the symmetry of the Raman active modes, M denotes a multi phono process, Dia is the peak for natural diamond, D and G are the D and G band of graphite, and trans-polyacetylene (t-PA) denotes the ω_1_ and ω_3_ vibration modes of trans-polyacetylene.

The synthesis of the structured non-conductive diamond film on Ti alloy is schematically illustrated in Figure 1a and based on prior research. The synthesis procedure relies heavily on colloidal chemistry. Briefly, the deposition process comprised of two successive seeding and CVD growth processes. In the first process, unfavorable seeding parameters (i.e., taking into account Zeta potential of DNDs and charge of substrate surface) were employed to afford sparse seeding of the DNDs on the Ti alloy surface. Subsequently, diamond was grown via the HFCVD process, yielding hemispherical grown diamond on the alloy. Afterwards, a second seeding and growth cycle was executed. However, these time-favorable seeding parameters were employed, affording high seeding densities. After the second growth process, a dense diamond coating was deposited on the whole sample, yet the hemispheres remained on the substrate surface, yielding in hierarchical roughness (micro/nano roughness), as shown in Figure 1b,c. The height and width of the hemispheres on the surface were 2.5 ± 0.2 µm and 3.5 ± 0.6 µm, respectively. The thickness of the continuous nanocrystalline diamond coating was around 200 nm (see also supporting information cross-sectional view of the diamond coating on Si, Figure S1). Successful coating of the Ti alloy was confirmed by Raman spectroscopy. Before coating with diamond, the Ti oxide (rutile phase) of the Ti alloy could be clearly detected, as Raman active modes with symmetries E_g_ and A_1g_ at 439 cm^−1^ and 603 cm^−1^, respectively, and the multi phonon process at 241 cm^−1^ were detected [42]. The existence of a (nanocrystalline) diamond coating on the Ti alloy was confirmed by Raman, as shown in Figure 1d. The generation of diamond was evidenced by the peak at 1333 cm^−1^. Furthermore, the peaks at 1350 cm^−1^ and 1560 cm^−1^ denote the D and G band of graphite, while the shoulder at 1148 cm^−1^ and the minuscule peak at 1460 cm^−1^ represent the ω_1_ and ω_3_ vibration modes of trans-polyacetylene (t-PA) [43,44]. t-PA is often formed as a byproduct during generation of nm-sized diamond crystals [45]. From the presence of these Raman bands, the generation of nanocrystalline diamond can be inferred [46].

### 3.2. Wettability of the Diamond Coatings (Toward Liquid Metal)

**Figure 2:**
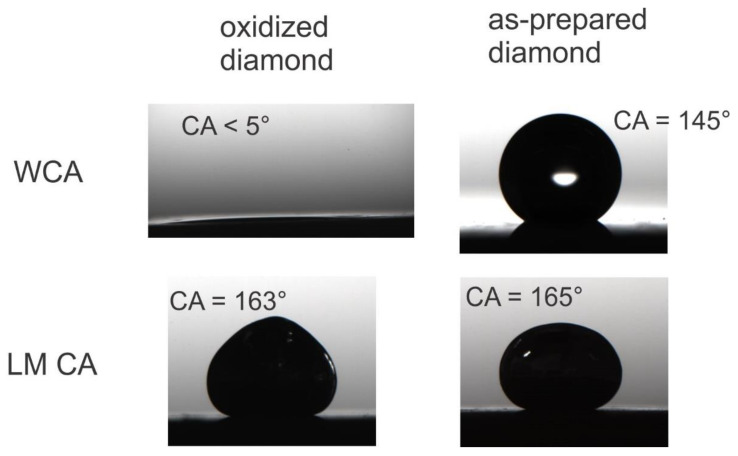
Water contact angle (WCA) and LM contact angle (LM CA) on oxidized and as-prepared rough diamond coating on Ti alloy.

Due to the reductive reaction regime in the CVD chamber, as-made diamond coatings are hydrophobic (H-terminated) [47,48]. The structured diamond coating we produced features (super)hydrophobicity with water contact angles (WCAs) around 150° due to a Cassie state and the presence of air pockets. One feature of diamond coatings is the ability to change the surface water wettability (surface free energy) without degradation of the integrity, morphology, or topography of the coating. By exposition to air plasma, the water contact angle was lowered to ≤ 5°, as shown in Figure 2. This change in WCA is attributed to the oxidation of the surface, yielding terminal groups such as –OH, =O, –O– and so on [47]. The change in surface termination also impacted the surface free energy (SFE) of the diamond coating, and the SFE determined via the van Oss method increased upon oxidation from around 50 mN/m to around 60 mN/m, while the contribution to this SFE changed substantially. The apolar Lifshitz-van der Waals γ_s_^LW^ interactions were nearly the exclusive contributor for the SFE of hydrophobic diamond coatings, while polar Lewis acid-based γ_s_^AB^ interactions can be detected for the oxidized diamond surface [47]. To evaluate the impact of surface free energy on the adhesion and wetting of liquid metal, liquid metal contact angles and roll-off angles were measured at first. The contact angles were determined to be 163.4 ± 2.0° and 162.5 ± 2.0° on the oxidized and H-terminated diamond coating, respectively. Thus, the liquid metal contact angle appeared to be independent on the surface free energy (surface termination), while it was impacted to some extend by surface roughness [29]. This might be the reason for the fact that, in the literature, the wetting and adhesion behavior of liquid metal was mostly attributed to surface roughness. The low impact of surface free energy on the LM contact angle might be attributed to the high yield stress [26], rendering deformation of the LM droplet upon changes in surface free energy challenging. Notably, deposited LM droplets could be removed swiftly by a tweezer or tilting the sample. Despite similar LM contact angles measured on both samples, the roll off angles differed significantly. For rolling off a LM droplet from the oxidized sample, a tilt angle of 11.3 ± 1.5° was necessary, while for the hydrophobic coating, a tilt angle of only 8.5 ± 1.0° was needed. This difference that can be ascribed to hydrophilic interactions between the hydrophilic oxide skin of the LM and the hydrophilic substrate was only possible for the oxidized diamond coating. Such hydrophilic interactions were already suggested to impact adhesion of LM [22,23], for example Gua et al. employed a coating based on a glue made of hydrophilic polymethacrylates (PMA) [24], which increased the adhesion of the LM toward various substrates. The obtained increased adhesion and wettability of the LM toward the surface was presumably due to hydrogen–bond interaction and lower roughness [24].

### 3.3. Comparison of Adhesion Resistance

In contrast to simple deposition techniques, such as contact angle and angle measurements, droplet impact measurements are more repeatable due to the fact that the former experiments rely heavily, for liquid metal (oxide), on the deposition procedure (care during deposition). Therefore, droplet impact experiments with varying impact heights were conducted (1–24 cm). The droplet velocity (V), which is dependent on the impact height (H) of the droplet and the droplet radius (R), can be estimated under the assumption that drag forces are negligible by Equation (1) [49].

(1) $$V=\sqrt{2g\left(H-R\right)}$$

The droplet diameter (D) can be determined by the mass (m) and the density (ρ) of the LM by Equation (2).

(2) $$D={\left(\frac{6m}{n\pi \rho }\right)}^{1/3}$$

The Weber number, a key dimensionless number, describes the ratio between inertial forces and stabilizing cohesive forces of a liquid. The Weber number (We) can be calculated by knowledge of the density, impact velocity, radius, and interfacial tension (liquid/gas) of the droplet, as shown in Equation (3) [50].

(3) $$We=\frac{\rho V^{2}R}{\gamma }$$

Due to the presence of the oxide skin for gallium-based liquid metals and the accompanying change in surface tension (interfacial tension), the full extent of which is unknown at this point [51], we assume for the calculation of the We that the surface tension does not change, which might underestimate the Weber number.

**Figure 3:**
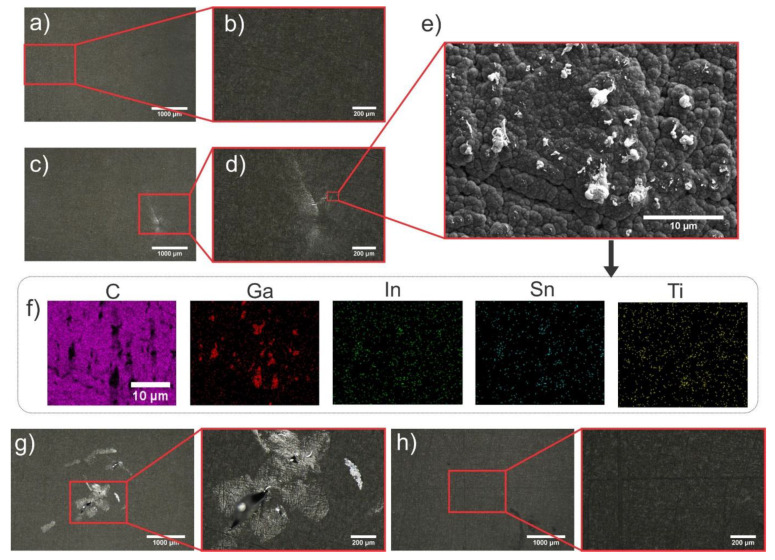
(**a**–**d**) Optical microscopy images of an oxidized rough diamond surface (coated on Ti, water contact angle ≤ 5°) after the LM droplet impact test. (**a**) Substrate surface after droplet impact from a height of 10 cm, and its magnification (**b**). (**c**) Substrate surface after droplet impact from a height of 24 cm and its magnification (**d**). (**e**) SEM micrograph of the rough diamond substrate after the droplet impact test (24 cm height, see (**c** and **d**)). (**f**) EDS maps (C, Ga, In, Sn, and Ti) of the sample, corresponding to (**c**) and (**e**). (**g**,**h**) Difference of the adhesion of LM on hydrophilic (**g**) and hydrophobic substrate (**h**) upon repeated LM deposition. Adhesion visible after 50 LM droplets deposited on hydrophilic substrate (**g**), while no adhesion is visible for the hydrophobic substrate after the same number of repetitions.

For low impact heights (<15 cm), the LM droplet could be readily removed and no residual LM could be detected with optical microscopy (see Figure 3a), while at impact heights equal or bigger than 15 cm residual LM could be found on both the (super)hydrophobic and (super)hydrophilic samples. By employing Equations (1) and (2), the radius of the droplet was calculated to 0.013 cm, yielding an impact velocity of 1.708 m/s and a We number of 4.11 at an initial height of 15 cm. We ascribe this wetting to a high peak impact force, overcoming the anti-adhesion properties of the coating. Here, the impact force may be comparable to the effect of forced wetting, which is often employed for patterning techniques [30,31,32,33,34,35]. In forced wetting approaches, the LM is pressed with a force toward the surface, yielding in better wettability and stronger adhesion. The LM oxide adhering to the rough substrates was found to be preferentially located at the surface asperities, which can be explained by higher pressure acting at these asperities, allowing a conformal contact between the rough LM oxide surface and the rough diamond coating [28]. The presence of the LM at the asperities was proven by SEM micrographs and EDS mapping, as shown in Figure 3e,f. Furthermore, we assumed that the adhesion is highly dependent on impact force, which in turn is dependent on impact velocity of the droplet and on the topography of the sample, while the surface free energy has only a minute impact on this.

Up to this point, the hydrophilic and hydrophobic surfaces showed a similar performance to repel LM (oxide). Similarly, in the literature, only a few examples discuss the relevance of surface free energy on anti-adhesion performance and found that surface free energy does not have an impact, even though intuitively it should have [29,52]. To further validate the impact of surface free energy on anti-adhesion performance, the long-term resistance of the surface toward LM (oxide) adhesion was investigated by repeated deposition and removal experiments. LM was deposited carefully on both the hydrophilic and hydrophobic samples up to 100 times. For the hydrophobic sample, no residual LM could be observed in microscopy images and, after the 100 deposition and removal cycles, the substrate was still LM lyophobic. In contrast, already after a few deposition and removal cycles, LM residue was visible by microscopy on the oxidized diamond sample. After around 50 depositions and retraction cycles, the LM droplet stuck to the diamond coating (see Figure 3g) and could not be removed with a tweezer, pipette, or by tilting the sample. We assumed that this effect was related to adhesion of micro/nano-patches of the LM oxide, induced by enhanced hydrophilic interactions, and evidenced by increases roll off angles (see above) between the oxidized LM and the hydrophilic substrate surface. These LM oxide patches were in contact with the rough surface and overcame the cohesion of the LM (oxide). As only a small ratio of LM oxide was in direct contact with a rough surface due to surface roughness and the previously discussed LM oxide roughness, the adhering LM patches denoted only a minute part of the total surface area. However, by repeated contact and retraction cycles, these patches grew and, at some point, dominated the wettability/adhesion of substrate. It should be noted that this adhesion upon repeated deposition might be an effect of exertion of force during the deposition and retraction cycles, though we were careful during this process. Therefore, we invite other researchers to investigate this phenomenon to ascertain its validity and extent, i.e., its effect on different coatings, substrates, and deposition forces.

A comparison of the behavior of the liquid metal in contact with the hydrophilic and hydrophobic surface is given in Table 1, showing at first glance similar behavior of the hydrophilic and hydrophobic diamond surface towards liquid metal wetting and adhesion. However, as detailed before, it shows different resistance toward liquid metal adhesion upon repeated liquid metal adhesion cycles, which will be exploited in the next section to enable enhanced interfacing of the liquid metal with the diamond surface.

### 3.4. Application of the Liquid Metal Adhesion to the Hydrophilic Diamond Coating

**Figure 4:**
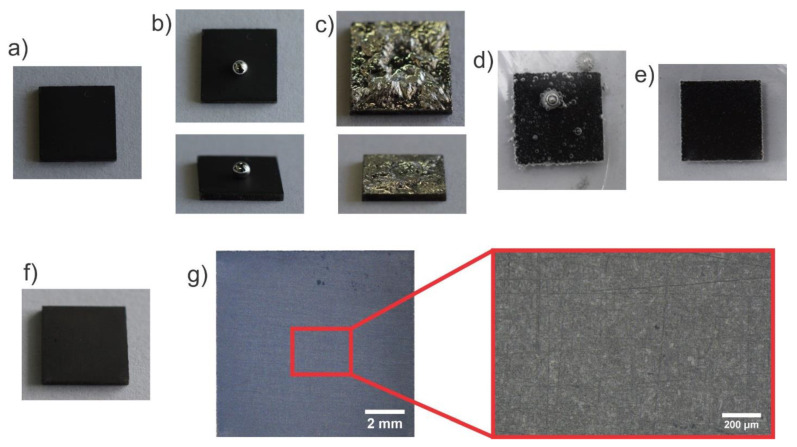
Application of the effect of adhesion of LM to diamond by repeated deposition and gentle force (massaging). The sample size is 10 mm × 10 mm. (**a**) Oxidized rough diamond coating (on Ti alloy) before liquid metal deposition. (**b**) Top and side view of a liquid metal deposited on the diamond coating, showing a high contact angle. (**c**) Optical image of the diamond coating with adhering liquid metal by applying the repeated deposition and massaging approach. (**d**) Removal of the liquid metal (oxide) from the surface of the diamond-coated Ti alloy by immersion in 1 mol/L H2SO4 solution. The liquid metal beads up due to removal of the oxide skin. (**e**) Second removal step in sulfuric acid. (**f**) Substrate after liquid metal removal with sulfuric acid and gentle rubbing with ethanol. (**g**) Optical microscopy images of the cleaned diamond coatings, signifying a clean and undamaged surface.

LM is often employed as an electric conductor in flexible electronics and a thermal conductor. In these applications, the good electric and thermal transport properties of the LM were exploited. Yet, for a good performance, several prerequisites had to be met. First, the LM interfaced with a material with high thermal and/or electrical conductivity, which were typically solid metals. However, solid metals are subject to corrosion by the LM, resulting in liquid metal embrittlement. This can be overcome by employing diffusion barriers [15,16,17,18]. On most of these diffusion barriers, LM showed poor wettability and adhesion, which limited the electric and thermal performance, i.e., the stability and so on. To improve the performance, a high contact area between the LM and the solid sample had to be established. Here, the adhesion of the LM upon repeated deposition on an oxidized surface could be exploited to obtain excellent interfacing of the LM with the solid sample. In Figure 4, the oxidized structured diamond coating on Ti is shown before and after the deposition of the LM by massaging the LM onto the substrate with a soft plastic syringe. An initially deposited LM droplet showed a high contact angle (Figure 4b). During massaging of liquid metal on the diamond surface, the liquid metal transitioned from a Cassie-Baxter state, stabilized due to trapped air and high yield stress of the liquid metal oxide, to a Wenzel state, where the liquid metal oxide was in contact with the cavities of the diamond coating. The final liquid metal (oxide) layer on the diamond coating appeared in the optical photograph to have some roughness, as shown in Figure 4c. This roughness could be related to the fabrication procedure, which was executed with an excess of liquid metal during massaging and subsequent removal of bulk liquid metal with a syringe, leaving excess liquid metal oxide wrinkling on the surface. Interestingly, the LM could be facilely removed by acid (i.e., hydrochloric acid, sulfuric acid, etc., at a concentration of 0.1 or 1 mol/L) or base without destroying the sample morphology, as shown in Figure 4, and even after adhering to the diamond coating for 4 days. Therefore, the removal process was anticipated to be independent on the duration of adhesion of the LM. The removal of the liquid metal was related to the removal of the oxide skin and the high interfacial tension of the “bare” liquid metal in these solutions, as published earlier [51,53,54]. Subsequently, the liquid metal could be removed facilely by tilting the sample and recycling it [54]. Afterwards, rare residual small beaded liquid metal located in the pitches between the surface protrusions was removed by gently rubbing and with subsequent rinsing with ethanol. Images during and after the cleaning steps are shown in Figure 4d,e, illustrating the facile and complete removal of the liquid metal from the surface. Similarly, the optical and microscopy images in Figure 4f,g illustrate the complete removal of the liquid metal from the surface. This cleaning step is enabled by the high chemical inertness, high mechanical robustness, and excellent adhesion of the diamond coating [40,41]. Moreover, in the view of the excellent tunability of diamond coatings, such as crystal size, electric conductivity, thickness of the coating, etc. [41], these coatings should have a great impact on liquid metal research in the future.

## 4. Summary and Conclusions

At first glance, the ability to withstand adhesion of liquid metal (oxide) was found to be independent on the surface free energy. For example, liquid metal contact angles on the hydrophilic and hydrophobic rough diamond coating were virtually the same. Similarly, the initial height for overcoming the anti-adhesion effect of the structured diamond surface upon droplet impact was the same for both surfaces. However, in contrast to previous results, we show that liquid metal may adhere to the (hydrophilic) structured surfaces upon repeated deposition/removal cycles, which we assigned to the generation of small adhering LM (oxide) patches at the surface asperities of the substrate, enabled by stronger interaction of the liquid metal oxide and the hydrophilic substrate surface, as well as gentle force (compare forced wetting approaches). The difference in wetting/adhesion of the liquid metal on these two structured diamond surfaces upon repeated depositions cycles with gentle force was exploited to achieve a high contact area between the liquid metal (wetting) and the diamond substrate. Finally, we showed that the wetting of the sample is completely reversible, the liquid metal can be removed from the surface by addition of acid or base, and the diamond-coated samples, as well as the liquid metal, can be recycled. Therefore, these diamond coatings are an interesting approach for stable interfacing of liquid metal with solid metal, especially taking into account the effect observed in this research project (increased adhesion). The observed effect may find application in flexible electronics and thermal management systems as a means to improve interfacing of the liquid metal with non-metal coatings.

## Figures and Tables

**Table 1:** Comparison of the roll-off angle, static contact angle, and number of deposition cycles for adhesion of the LM on hydrophilic and hydrophobic rough diamond coatings on Ti alloy.

Measurement Method	Oxidized Diamond (WCA ≤ 5°)	H-Terminated Diamond (WCA ≈ 150°)
Scroll off angle	11.3 ± 1.5°	8.5 ± 1.0°
Minimum height (speed, Weber number (We)) for adhesion of LM upon droplet impact	15 cm (1.7 m/s, We 4.11)	15 cm (1.7 m/s, We 4.11)
Number of deposition/removal cycles necessary for LM adhesion	53.3 ± 2.5	>100
(static) contact angle	163.4 ± 2.0°	162.5 ± 2.0°

## Supplementary Materials

The following are available online at https://www.mdpi.com/1996-1944/13/10/2283/s1, Figure S1: Cross-sectional SEM micrographs of the diamond coating on Si.
