# Supplementary material for: Is There a Relationship between Surface Wettability of Structured Surfaces and Lyophobicity toward Liquid Metals?
Source: Materials (Basel). 2020 May 15;13(10):2283. doi: 10.3390/ma13102283 (PMC7288057; doi:10.3390/ma13102283)
Supplement: Supplementary file 1 [file materials-13-02283-s001.pdf]

materials

Article

Is There a Relationship between Surface Wettability of Structured Surfaces and Lyophobicity toward Liquid Metals?

Stephan Handschuh-Wang [1,*], Lifei Zhu [1], Tiansheng Gan [1] and Tao Wang [2]

[1] College of Chemistry and Environmental Engineering, Shenzhen University, Shenzhen 518055, China; 1800221021@email.szu.edu.cn (L.Z.); gantiansheng@szu.edu.cn (T.G.)
[2] Functional Thin Films Research Center, Shenzhen Institutes of Advanced Technology, Chinese Academy of Sciences, Shenzhen 518055, China; tao.wang1@siat.ac.cn
* Correspondence: stephan@szu.edu.cn; Tel.: (+86)-755-2653-6627

Received: 20 April 2020; Accepted: 13 May 2020; Published: date

Supporting information

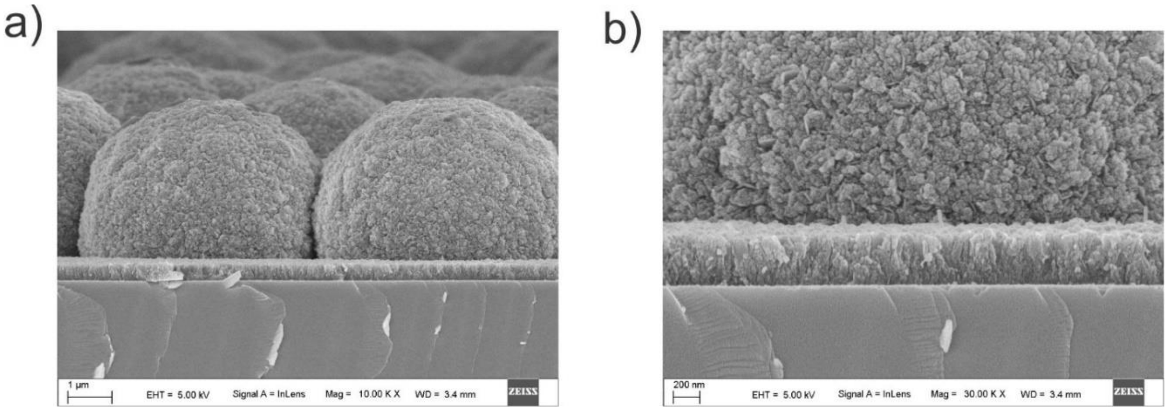

Figure S1. Structured diamond coating on Si. SEM cross-sectional view of the diamond coating (**a**) and its magnification (**b**).

© 2020 by the authors. Submitted for possible open access publication under the terms and conditions of the Creative Commons Attribution (CC BY) license (http://creativecommons.org/licenses/by/4.0/).
